# Production and Characteristics of a Novel Xylose- and Alkali-tolerant GH 43 β-xylosidase from *Penicillium oxalicum* for Promoting Hemicellulose Degradation

**DOI:** 10.1038/s41598-017-11573-7

**Published:** 2017-09-14

**Authors:** Yanxin Ye, Xuezhi Li, Jian Zhao

**Affiliations:** State Key Laboratory of Microbial Technology, Shandong University, Jinan, 250100 P.R. China

## Abstract

β-xylosidase is a pivotal enzyme for complete degradation of xylan in hemicelluloses of lignocelluloses, and the xylose- and alkali-tolerant β-xylosidase with high catalytic activity is very attractive for promoting enzymatic hydrolysis of alkaline-pretreated lignocellulose. In this study, a novel intracellular glycoside hydrolase family 43 β-xylosidase gene (*xyl43*) from *Penicillium oxalicum* 114-2 was successfully high-level overexpressed in *Pichia pastoris*, and the secreted enzyme was characterized. The β-xylosidase Xyl43 exhibited great pH stability and high catalytic activity in the range of pH 6.0 to 8.0, and high tolerance to xylose with the Ki value of 28.09 mM. The Xyl43 could effectively promote enzymatic degradation of different source of xylan and hemicellulose contained in alkaline-pretreated corn stover, and high conversion of xylan to xylose could be obtained.

## Introduction

Xylan is the main component of hemicelluloses in lignocellulosic materials, especially in agro-industrial residues, such as sugarcane bagasse, corn stover, etc. comprising 15–35% of each plant’s dry weight^1^. Xylan is a heterogeneous polysaccharide consisting of a backbone of β-1,4-xylose units that are attached by side chains such as arabinose, methyl glucuronic acid, and acetate groups^2^. A series of enzymes is essential when xylan is completely degraded to monosaccharide, mainly including endo-β-xylanases (EC 3.2.1.8) and β-D-xylosidases (EC 3.2.1.37). β-Xylosidases play crucial role in enzymatic degradation of hemicelluloses. Endo-β-xylanases cleave the main chain of xylan and liberate xylo-oligosaccharides with diverse degree of polymerization, and then, xylo-oligosaccharides are ultimately degraded to xylose by β-D-xylosidases^3^. To effectively hydrolyze xylan and accumulate high levels of monosaccharides in the enzymatic hydrolysis process for lignocelluloses, the β-xylosidase should have high tolerance for glucose and xylose, that is to say, exhibit high Ki values for glucose and xylose. On the other hand, high activity β-xylosidase is also important for the enzyme application in industry to decrease enzyme dosage and production cost. Up to now, however, β-xylosidases in most commercial enzyme cocktails are deficient^4, 5^, and most β-xylosidases from microorganisms are sensitive to xylose and are inhibited with the Ki values as low as 2–10 mM^6^. So, to produce β-xylosidase with high xylose-tolerance and high catalytic activity is very important for enhancing enzymatic hydrolysis of hemicellulose and bioconversion of lignocelluloses.

β-xylosidases are divided into families 3, 30, 39, 43, 52 and 54 of glycoside hydrolase (GH) based on their amino acid sequence similarities^7^. So far, most of fungal β-xylosidases reported in literature belong to GH families 3, 43 and 54. Members of GH 3 and GH 54 employ retaining catalysis and have two catalytic residues (a aspartate and a glutamate), while GH 43 β-xylosidase has inverting catalysis and possesses three catalytic residues (two aspartates and a glutamate). The third catalytic residue, aspartate, is supposed to be involved in the adjustment of the pKa of the general acid and the adjustment of the correct orientation of both proton donor and substrate^8, 9^. Enzymatic reaction of inverting GH 43 does not perform a nucleophilic attack through an enzyme-bound intermediate susceptible, which benefits GH-catalyzed hydrolysis^10^. The GH 43 β-xylosidase does not also show transglycosylation activities at high substrate concentrations^11^. These distinct features afford them an effectively prevail over retaining enzymes that proceed through two transition states and can be highly active in catalyzing transglycosylation reactions that compete against hydrolysis^12^. Thus, the GH 43 β-xylosidases are greatly promising candidates of high-efficiency β-xylosidases for the degradation of plant biomass.

**Figure 1:**
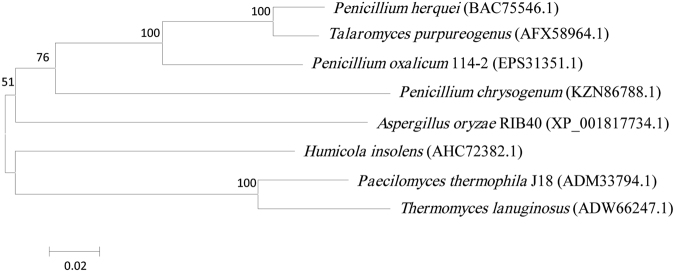
Phylogenetic tree analysis of Xyl43 from *Penicillium oxalicum* 114-2 with other known GH 43 fungal β-xylosidases. Numbers in brackets indicate the GenBank accession numbers.

Compared with GH 3 β-xylosidases, only a few GH 43 β-xylosidases from filamentous fungi (Fig. 1) were described and characterized now. Most of the known fungal β-xylosidases from GH 43 were greatly tolerant to xylose, for example, Xyl43A and Xyl43B with the Ki values of 79 mM and 292 mM from *Humicola insolens* respectively^6^, TlXyl43 with the Ki value of 63 mM from *Thermomyces lanuginosus* ^13^, PtXyl43 with the Ki value of 139 mM from *Paecilomyces thermophile* ^14^. These β-xylosidases without predicting signal peptides were expressed in *Escherichia coli* (*E*. *coli*) and large quantities of proteins (except for Xyl43B) secreted into the medium, however, efficient secretion of the recombinant proteins into the culture medium of *E*. *coli* remains a challenge due to the intrinsic limitations of the secretion machinery^15, 16^. For the expression of heterologous proteins such as fungal enzymes, yeasts have many advantages which benefit industrial processes and the environment, for example, no optimizing codon of genes, the ability to perform protein folding, post-translational modifications, and high-level production of recombinant proteins secreted into fermentation media, etc. So yeasts are good choices and preferred over bacterial expression systems^7^. Up to now, however, limited GH 43 β-xylosidases from filamentous fungi were expressed in yeasts. It was reported that GH 43 β-xylosidase from *Paecilomyces thermophila* was expressed in *Pichia pastoris* (*P*. *pastoris*) and secreted into the culture medium with the protein concentration of 0.22 mg/l^17^, but the low level production of secretion protein did not meet the criteria for potential industrial applications. Therefore, it is still challenge to obtain hyper-production and high xylose-tolerant GH 43 β-xylosidases that secreted into the extracellular for optimizing hemicellulase cocktails and promoting thorough degradation of xylan to xylose.

*Penicillium oxalicum* 114-2 isolated in our laboratory is a fast growing filamentous fungus and secretes a variety of lignocellulolytic enzymes^18^. The genome of the strain had been sequenced (Accession number GCA_000346795.1 in GenBank), and a novel gene sequence encoding a putative GH 43 β-xylosidase was found on the basis of the *P*. *oxalicum* genomic DNA sequence. For GH 43 β-xylosidases distinct enzymatic properties and its great potential applications in degradation of hemicellulose, we try to heterologous express the putative GH 43 β-xylosidase gene *xyl43* from *P*. *oxalicum* 114-2 in *P*. *pastoris* X-33 and characterize the enzyme for its industrial applications. The potential application of the GH 43 β-xylosidase in enzymatic hydrolysis of hemicellulose in lignocellulosic biomass was also evaluated by using different sources xylans and NaOH-pretreated corn stover.

## Results and Discussion

### Cloning and sequence analysis of gene *xyl43*

According to the genome sequences of the *P*. *oxalicum* 114-2, a putative gene encoding β-xylosidase (GenBank: EPS31351.1) was found. The gene fragment of *xyl43* was shown 1044 bp which was obtained with primers xyl-f and xyl-r using the cDNA as template. Comparison of the genomic DNA and cDNA sequence, there were not introns in the encoding gene of *xyl43*. This case also similar to the β-xylosidase genes derived from other filamentous fungus^19, 20^. The deduced Xyl43 was encoded 348 residues with a theoretical molecular weight of 38.61 KDa, which is consistent with others of β-xylosidases from *Paecilomyces thermophile* ^17, 20^. The signal peptide had not been predicted at N-terminal of deduced Xyl43 using the Signal P 4.1 Server (http://www.cbs.dtu.dk/services/SignalP/). It was reported that there were ten and twelve potential N-glycosylation sites in sequences of β-xylosidase from *Aspergillus* sp. BCC125 and *Phanerochaete chrysosporium*, respectively^21, 22^. But only one possible N-glycosylation sites and seven potential O-glycosylation sites were found in the Xyl43 sequence using the NetNGlyc1.0 server (http://www.cbs.dtu.dk/services/NetNGlyc/) and the program NetOGlyc 4.0 server (http://www.cbs.dtu.dk/services/NetOGlyc/) (supplementary Table S1).

Homologous amino acid sequences of the deduced Xyl43 with other known proteins were searched in GenBank using the BLAST server. According to the sequence alignment of the Xyl43 from *P*. *oxalicum* with other reported GH 43 β-xylosidases from fungus (supplementary Fig. S1), the phylogenetic tree was constructed using MEGA4.0 Software (Fig. 1), and found that the Xyl43 shared apparently close relationship with the β-xylosidases from *Penicillium herquei* and *Talaromyces purpureogenus*. The enzyme showed a high degree of similarity to the conserved sequences of glycoside hydrolases belonging to GH family 43 and exhibited a significant similarity to β-xylosidases or β-xylosidases/α-L-arabinofuranosidases which experimentally verified from *Penicillium herquei* (88% of identity, E value: 0.0), *Talaromyces purpureogenus* (87% of identity, E value: 0.0), *Penicillium chrysogenum* (75% of identity, E value: 0.0), *Humicola insolens* (73% of identity, E value: 2e-179), *Thermomyces lanuginosus* (72% of identity, E value: 0.0), *Paecilomyces* sp. J18 (74% of identity, E value: 0.0), and *Aspergillus oryzae* RIB40 (73% of identity, E value: 0.0). It was also found by i-TASSER alignment that secondary structure of the Xyl43 (supplementary Fig. S1) highly matched with other known secondary structure of GH43 in PDB (supplementary Table S2), and was coincidence with the secondary structure of GH 43 β-xylosidase/α-arabinofuranosidase (PDB code: 5 gllA, 57% amino acid identity, E-value: 4.59e-113) from a compost microbial metagenome. Moreover, homologous searching of the protein crystal structure using the PDB database revealed that Xyl3A highly matched the tertiary structure of RS223-BX (59.74% of identity), which was cloned from an anaerobic mixed microbial culture^23^ and it was predicted the tertiary structure of Xyl43 as an available template.

### Expression and purification of β-xylosidase Xyl43

**Figure 2:**
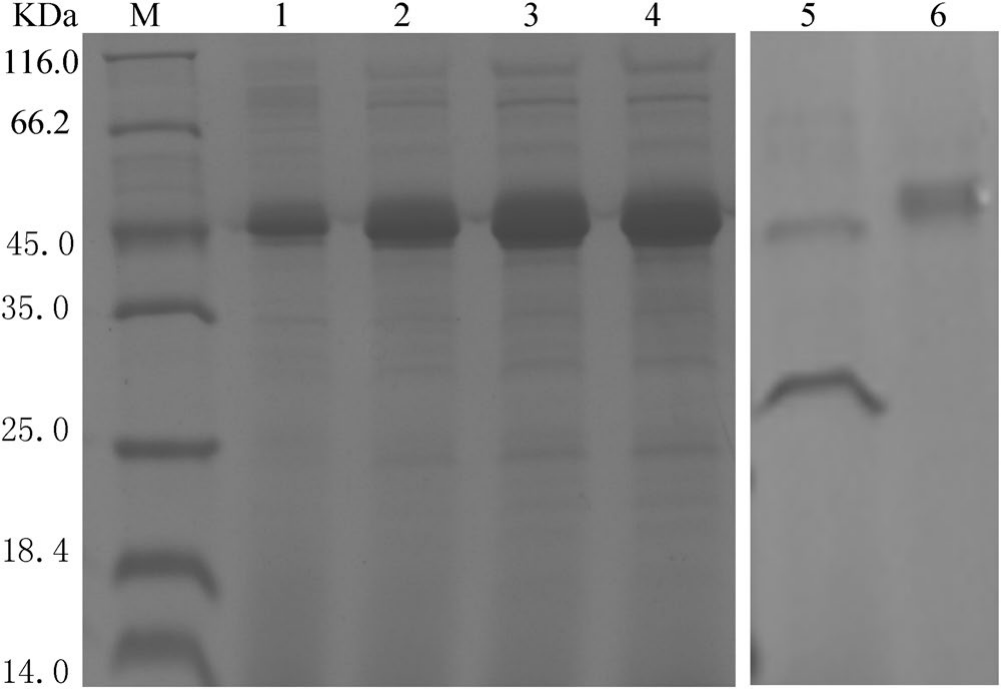
SDS-PAGE analysis of Xyl43. Lane M, protein marker; Lane 1–4, culture supernatants after 1, 2, 3, 4 days, respectively; Lane 5, Xyl43 after Endo H treatment; Lane 6, purified Xyl43. The lanes 5 and 6 were cropped from the supplementary Fig. S4.

Xyl43 with a α-factor secretion signal peptide in a secretory mode was successfully expressed in host *P*. *pastoris* X-33. Some positive transformants were randomly picked up and cultivated on YPDS plates at 30 °C for 60 h with different zeocin concentrations (200~500 μg/ml) for screening multi-copy positive transformations. A strain with multi-copy that resisted 250 μg/ml zeocin was obtained (Supplementary Fig. S2) and incubated in 500 ml shaking flask at 30 °C, 200 rpm for producing the recombinant Xyl43. After incubation of 4 days (Fig. 2), the Xyl43 protein concentration in culture supernatant reached approximately 92.8 mg/l (supplementary Fig. S3). The expression level of the Xyl43 was higher than that of GH 43 β-xylosidase (0.22 mg/l) from *Paecilomyces thermophila* ^17^ and GH 3 β-xylosidase (32 mg/L) from *Neurospora crassa* which were expressed in *P*. *pastoris* ^24^, but slightly lower than that (100 mg/l) of both GH 3 β-xylosidases from *Aspergillus oryzae* and *Humicola insolens* Y1 which were expressed in *P*. *pastoris* ^25, 26^. In our knowledge, so far, it is the highest production of fungus GH43 β-xylosidase expressed in *P*. *pastoris* in literatures.

The crude Xyl43 was purified using the HisTrap^TM^ FF column. The purified Xyl43 exhibited a specific activity of 4.96 IU/mg under the optimum conditions. The molecular mass is about 45 kDa based on the SDS-PAGE analysis (Fig. 2), which higher than the calculated molecular mass of the protein, probably due to glycosylation. Huy *et al*. reported the molecular mass of Endo H-treated recombinant β-xylosidase expressed in *P*. *pastoris* was obviously decreased by about 17 KDa, may be due to the twelve potential N-glycosylation sites in the β-xylosidase sequence^22^. However, the molecular mass of the Xyl43 had a slight decrease after deglycosylation with Endoglycosidase H (Fig. 2), this result was presumably caused due to the presence of only one potential N-glycosylation site of Xyl43. The protein was removed from SDS-PAGE and verified to be β-xylosidase from *P*. *oxalicum* using mass spectrometry (data no shown).

### Enzymatic characteristics of purified recombinant Xyl43

#### Optimal pH and stability on pH value

**Figure 3:**
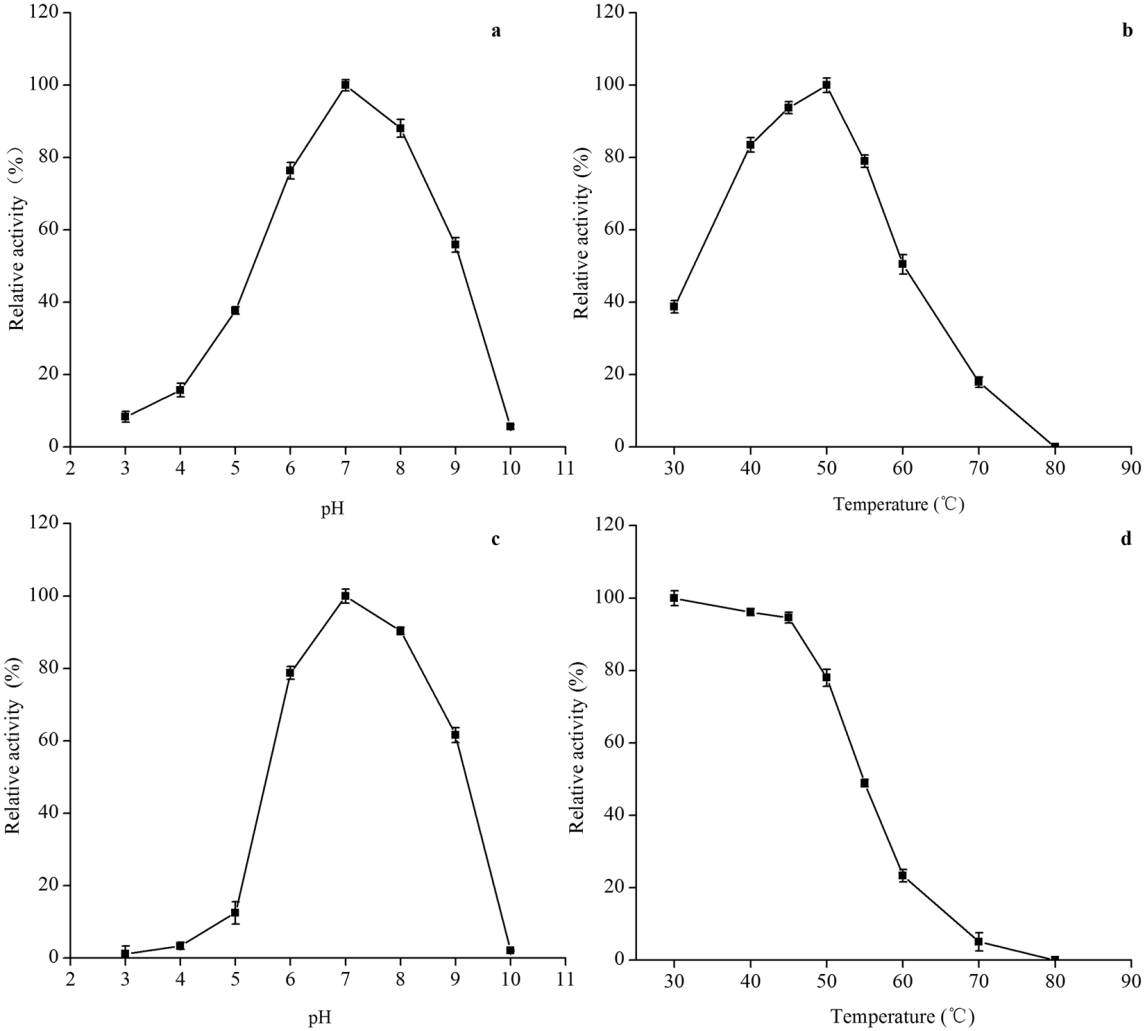
Characterization of purified β-xylosidase Xyl43. (**a**) Effect of pH on activity. (**b**) Effect of temperature on activity. (**c**) Stability in different pH. (**d**) Stability in different temperatures. The highest enzyme activity was set to 100%.

The effects of pH on activity of the β-xylosidase Xyl43 were detected. Generally, suitable pH of most reported β-xylosidases from fungi was in the acidic pH range of 3.0–6.0, though a few β-xylosidases are known to exhibit activity in the alkaline range of pH (Table 1). It was found that the optimum pH for the Xyl43 was pH 7.0 at 50 °C (Fig. 3a), and retained 76.35% and 55.85% of catalytic activities at pH 6.0 and 9.0, respectively. It was relatively stable in the range of pH 6.0 to 9.0, retaining more than 61% residual activity (Fig. 3c). Compared to the β-xylosidases reported from *Penicillium sp*. strains^19, 27^ and other fungal species^21, 24, 28^, this stability of the Xyl43 in the neutral and alkaline pH value was very important because it can be directly used in enzymatic hydrolysis process of neutral- and alkaline- pretreated agro-residues for high efficiency producing fermentable sugars by the synergy with alkali-xylanase^29^.

**Table 1:** Summarized enzymatic properties of some β-xylosidases reported in literatures and the Xyl43 in this study.

Fungus	GH family	Molecular mass	Optimum pH	Optimum Temperature (°C)	Km (mM)	Vmax (µmol/min/mg)	Specific activity (IU/mg)	Ki (mM)	References
*Penicillium oxalicum* 114-2	43	45^a^	7.0	50	4.05	88.82	4.96	28.9	This work
*Paecilomyces thermophila* J18	43	39.31^a^	7.0	60	8.0	54	0.26	—	17
*Neurospora crassa*	3	81.8^a^	5.0	50	8.9	1052	—	1.72	24
*Aspergillus oryzae*	43	84.7^a^	4.5	55	1.0	250		2.72	25
*Thermomyces lanuginosus*	43	52.3^a^	7.0	50	—	—	2.29	—	48
*Penicillium purpurogenum*	43	47.31^c^/37.07^a^	5.0/6.0	−/40	12/0.55	−/−	−/−	−/−	19, 49
*Phanerochaete chrysosporium*	43	83.0^a^	5.0	45	12.70	2812	1797	10	22
*Humicola insolens* Y1	3	83.2^a^	6.0	60	2.51	37.33	11.6	29.0	26
*Humicola insolens* Y1	43	62.0^b^	7.0	50	1.29	2.18	1.7	292	6
*Humicola insolens* Y1	43	37.0^b^	6.5	50	12.2	203.8	20.5	79	6
*Thermomyces lanuginosus*	43	51.6^b^	6.5	55	3.90	107.6	45.4	63	13
*Paecilomyces thermophila*	43	52.3^b^	7.0	55	4.50	90.2	45.4	139	20
*Aspergillus oryzae*	3	—^b^	7.0	30	0.48	42.6	6.1	—	30
*Trichoderma reesei*	—	100^c^	4.0	60	0.08	—	—	2.3	50

^a^Expressed in *P*. *pastoris*; ^b^Expressed in *E*. *coli*; ^c^Purified from native fungi.

#### Optimal temperature and thermo-stability

The β-xylosidase Xyl43 exhibited the highest activity at 50 °C at optimal pH 7.0 (Fig. 3b). It was stable at below 50 °C, but the activity rapidly decreased when the temperature above 50 °C (Fig. 3d). The thermo-stability of the Xyl43 was similar to reported β-xylosidases from mesophilic fungus^19, 24, 30^.

#### Effect of different monosaccharide sugars on activity

**Figure 4:**
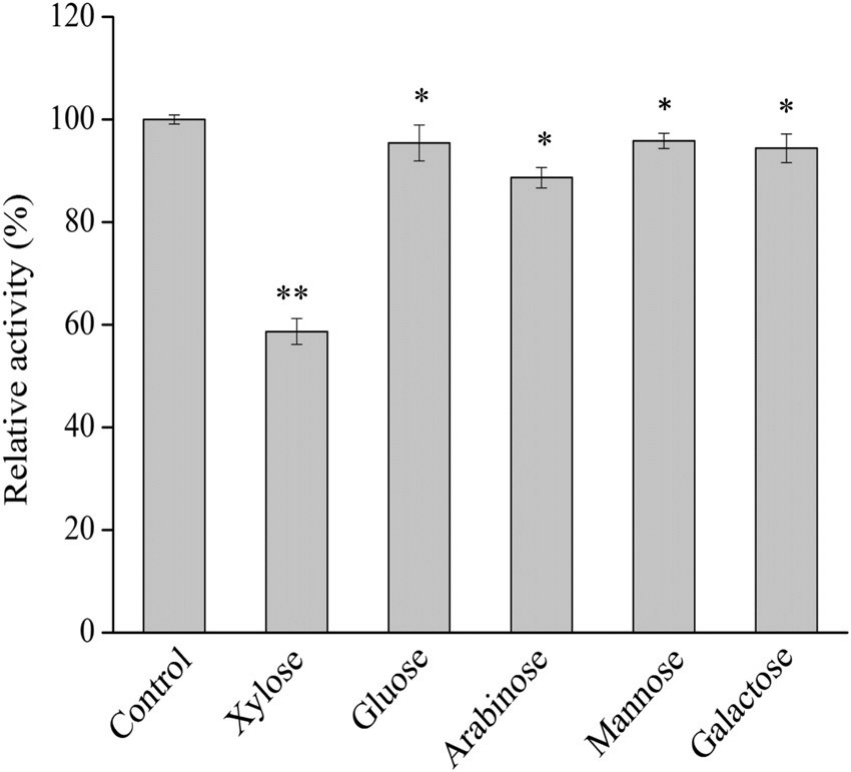
Effect of different monosaccharide sugars in reaction system on the purified Xyl43 activity. All reactions were carried out at optimal pH (7.0) and temperature (50 °C) for 30 min using 1 mg/ml of *p*NPX as substrate. Control reactions were conducted without any addition of monosaccharide sugars under identical conditions. The data marked with ** and * respectively represented there was significant difference (p < 0.01) and not significant difference (p > 0.05).

High tolerance of the enzyme on monosaccharide sugars is very important for producing the hydrolysis liquor with high consistency fermentable sugars, which means that high content end products could be obtained by following fermentation process and production cost. The effect of different monosaccharide sugars on the activity of Xyl43 was researched and found that glucose, mannose and galactose at the 20 mM consistency did not affect the catalytic activity of the Xyl43 enzyme (Fig. 4). The arabinose of 20 mM has a slightly inhibition on the Xyl43 catalytic activity (decreased to 88.7% of initial activity), which was similar to inhibition on activity of β-xylosidase from *P*. *woosongensis* at a concentration of 10 mM^31^. But xylose exhibited a strong suppression on the Xyl43 activity (decreased about 40%).

As xylan is a main component of hemicelluloses, especially in agro-residues such as corn stover, the effect of various xylose concentrations on Xyl43 activity was investigated and a Dixon plot was constructed on the basis of the experimental results. It was reported that fungal β-xylosidases, e.g., from *Neurospora crassa* ^24^, *Talaromyces emersonii* and *Trichoderma reesei* ^32^, *Aspergillus oryzae* ^25^, *Penicillium sclerotiorum* ^33^, *Aspergillus niger* ^34^, are sensitive to xylose and the Ki values are between 2 mM and 12 mM. It was found by the Dixon plot that the purified Xyl43 has a Ki value of 28.09 mM (Table 1), showed high tolerability on xylose, similar to xylose-tolerance β-xylosidases from *Humicola insolens* ^26^ and *Aspergillus nidulans* ^35^. Compared with other β-xylosidases reported in literatures, high xylose tolerance of the GH 43 Xyl43 was very beneficial for the enzyme application in the bioconversion of xylan-rich biomass such as corn stover, hardwood, etc.

#### Effect of metal ions and chemical reagents on activity

**Figure 5:**
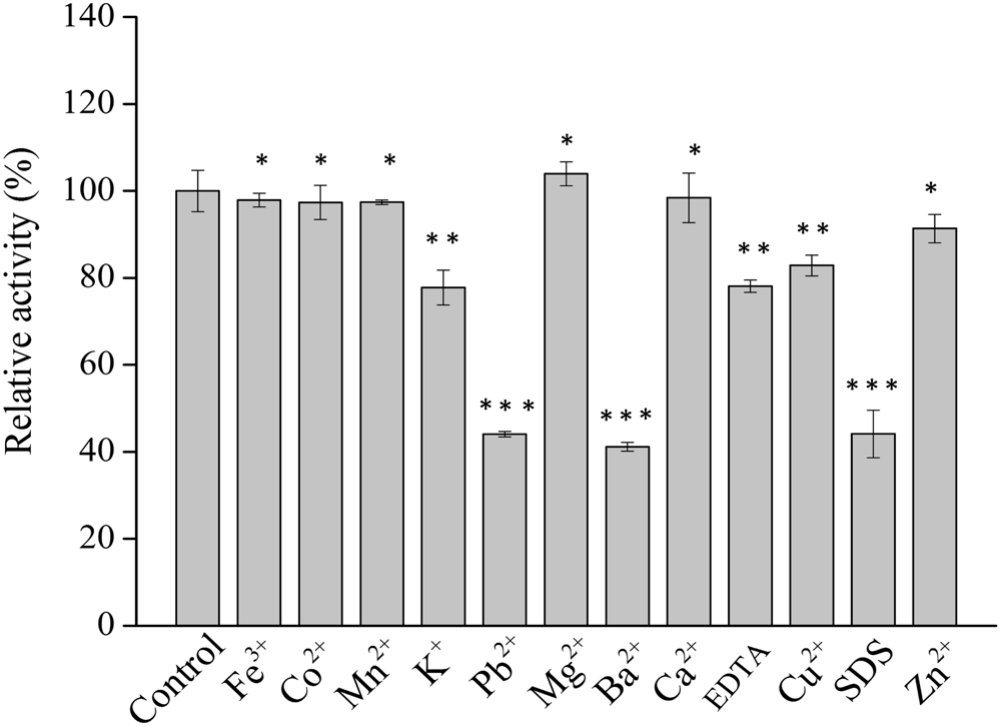
Effect of different metal ions and chemical reagents at 5 mM consistency on the purified Xyl43 activity. All reactions were incubated at optimal pH (7.0) and temperature (50 °C) for 30 min using 1 mg/ml of *p*NPX as substrate. Control experiments were done in the absence of metal ions and chemical reagents under the same reaction conditions. The data marked with ** and *** showed there were significant difference (p < 0.05) and specially significant difference (p < 0.001) respectively, and an asterisk * means that the difference was not significant (p > 0.05).

The influence of various metal ions and chemical reagents on the activity of Xyl43 was also investigated and shown in Fig. 5. The enzymatic activity of Xyl43 was slightly enhanced to 103.9 ± 3.1% by Mg^2+^, which similar to the β-xylosidase from *P*. *thermophile* (106.7 ± 3.1%)^14^, possible because the Mg^2+^ may activate and protect the active centre of β-xylosidases. The ions of Pb^2+^ and Ba^2+^and SDS severely inhibited the Xyl43 activity. It was reported that the β-xylosidase from *Geobacillus theodenitrifican* also strongly inhibited by Pb^2+^ and SDS at 5 mM consistency^36^, however, addition Pb^2+^ or Ba^2+^ did not affect the enzyme activity of Xyl3A from *Humicola insolens* Y1^26^ and β-xylosidase from *Aureobasidium* ^37^. In addition, Cu^2+^, K^+^ and EDTA moderately (<20%) affect the Xyl43 activity in this study, similar results had been reported for the β-xylosidases from *P*. *woosongensis* ^31^. The presence of Mn^2+^, Ca^2+^, Fe^3+^, Co^2+^ and Zn^2+^ had no effects on the Xyl43 activity, whereas these metal ions strongly inhibited the β-xylosidase from *P*. *chrysosporium* ^22^.

#### Substrate specificity and kinetic constants of Xyl43

To investigate the substrates specificity of the β-xylosidase Xyl43, different substrates, for example, *p*NPG, *p*NPX, *p*NPAf, *p*NPC, beechwood xylan, glucan, mannan, polygalactose and arabinan, were used. It was found that the Xyl43 did not act on these substrates except for *p*NPX, which similar to the β-xylosidase from *P*. *herquei* ^27^, indicated that the Xyl43 has unique substrate specificity.

Based on the Lineweaver-Burk plots, the calculated Km and Vmax of the Xyl43 using the *p*NPX as substrate were 4.05 mM and 88.82 μmol min^−1^ mg^−1^, respectively. Table 1 showed the Xyl43 had significantly lower Km values than the β-xylosidases from *Humicola insolens* (12.2 mM)^6^, *Paecilomyces thermophile* (8 mM)^17^, *Neurospora crassa* (8.9 mM)^24^, but similar to the β-xylosidases reported from *Thermomyces lanuginosus* and *Paecilomyces thermophile* ^13, 20^. Moreover, this recombinant Xyl43 exhibited higher Vmax toward *p*NPX than that of some β-xylosidases reported. When *p*NPX containing 10 mM xylose was used as substrate, the Vmax value was altered to 54.33 μmol min^−1^ mg^−1^, but Km value almost no changed (4.85 mM), suggesting the xylose, which is the end-product of the xylan, was an uncompetitive inhibitor which reduced the Vmax but did not affect the Km.

### Potential of the Xyl43 in enzymatic hydrolysis of hemicelluloses xylans and alkaline pretreated corn stover

To evaluate the potential of the Xyl43 in degradation of hemicellulose, enzymatic hydrolysis of various xylans that from corncob, beechwood, and sugarcane bagasse respectively and alkaline pretreated corn stover (APCS) were conducted using different emzymes such as the β-xylosidase Xyl43, purified endo-xylanase (Xyn), crude xylanase RE-10 or their mixtures.

Commonly, β-xylosidases mainly hydrolyse xylo-oligosaccharides from the non-reducing end to produce xylose. Table 2 showed that only negligible amounts of xylose released when the purified Xyl43 was added alone to the hydrolysis system of three types of xylans, which should due to a limited amount of non-reducing ends in the xylans. When employing Xyn alone, low amounts of xylose released, but the hydrolysis efficiencies of all the xylans were obviously enhanced by using treatment with the Xyl43 together with the Xyn, and xylose released increased by 350.81%, 357.96% and 69.7% for corncob xylan, beechwood xylan and sugarcane bagasse xylan, respectively. All the results showed synergism effects occurs between Xyl43 and Xyn during hydrolysis of the different types of xylans, in which, xylan was degraded to xylo-oligosaccharides by the endo-xylanase Xyn and then effectively cleft to xylose by the Xyl43. The synergy factors was calculated and showed that they are 4.14, 4.43 and 1.70 for corncob xylan, beechwood xylan, and sugarcane bagasse xylan, respectively. Kirikyali *et al*. also reported that the combination of xylanase and β-xylosidase enhanced the reducing sugar release from xylan compared with the treatment with β-xylosidase or xylanase alone^38^. Compared to the corncob xylan and beechwood xylan, the hydrolysis degree of sugarcane bagasse xylan was low, should due to the existence of branching hetero-substituents, which may hinder enzyme action on substrate^39^.

**Table 2:** Enzymatic hydrolysis of various isolated xylans using different enzymes^a^.

Substrates	Enzymes	Dosages (IU/mg, DW)	Xylose concentration in hydrolysis liquor (mg/ml)	Conversion of xylan to xylose (%)	Synergy factor
Corncob xylan	Xyn	0.20	0.62 ± 0.01	16.21 ± 0.24	—
	Xyl43	0.05	0.05 ± 0.09	1.43 ± 2.38	—
	Xyn + Xyl43	0.20 + 0.05	2.77 ± 0.28	73.08 ± 7.34	4.14
Beechwood xylan	Xyn	0.20	0.54 ± 0.07	14.26 ± 1.85	—
	Xyl43	0.05	0.04 ± 0.06	1.14 ± 1.58	—
	Xyn + Xyl43	0.20 + 0.05	2.58 ± 0.04	68.14 ± 1.06	4.43
Sugarcane bagasse xylan	Xyn	0.20	0.47 ± 0.05	12.30 ± 1.85	—
	Xyl43	0.05	0	0	—
	Xyn + Xyl43	0.2 + 0.05	0.79 ± 0.09	30.88 ± 2.38	1.70

^a^Hydrolysis condtions: 50 °C, pH 7.0 for 24 h.

Alkaline pretreatment is one of common pretreatment methods for enhancing enzymatic hydrolysis of lignocellulosic materials, which can remove lignin from biomass, but more hemicelluloses were retained in pretreated biomass. In this study, alkaline pretreated corn stover (APCS), which mainly consist of 49.81% glucan, 19.77% xylan and 10.84% lignin, was used as substrate for assessing the potential of the Xyl43 in enzymatic hydrolysis of APCS (Table 3). It was found that, similar to separated xylans, enzymatic hydrolysis of APCS using the Xyl43 alone just released negligible amounts of xylose too, and hydrolysis of APCS using the single Xyn or crude xylanase RE-10 produced xylose of 0.42 mg/ml and 0.33 mg/ml respectively. When only 0.25 IU/g DW of the Xyl43 was added to the reaction mixture together with Xyn or RE-10, the production of xylose markedly increased to 0.81 mg/ml and 1.43 mg/ml xylose respectively, and the calculated synergy factors for the release amount of xylose were 1.81 between Xyl43 and Xyn and 3.96 between Xyl43 and RE-10, respectively. The yield of xylose increased along with the increasing of Xyl43 dosage. The conversion of xylan was increased to 11.81% and 16.53% using the mixtures of Xyl43 + Xyn and Xyl43 + RE-10 when dosage of Xyl43 was 1.0 IU/g DW respectively, and the synergy factors for the release amount of xylose were 2.76 and 4.89, respectively. The low xylose yields from APCS by synergistic action of Xyl43 with Xyn (or RE-10) were due to the low dosages of Xyl43, Xyn and RE-10 and no addition of cellulase. In addition, the steric hindrance of the high cellulose content in the substrate (49.81%) could also result in the low xylose yields. But this results still suggested that the Xyl43 can effectively enhanced hydrolysis of xylan remained in APCS and increased yield of xylose by synergistic effect with Xyn or RE-10, showed a good potential in degradation of hemicellulose for bioconversion of hemicellulose in biomass. Table 3 also showed that the mixture of Xyl43 and RE-10 was more effective than that of Xyl43 and Xyn, possible because that crude xylanase RE-10 was a hemicellulase cocktails, and addition of the Xyl43 further optimized the hemicellulase cocktails for promoting degradation of hemicellulose.

**Table 3:** Enzymatic hydrolysis of xylan remained in APCS using different enzymes^a^.

Substrates	Enzymes	Dosages (IU/g, DW)	Xylose concentration in hydrolysis liquor (mg/ml)	Conversion of xylan to xylose (%)	Synergy factor
APCS	Xyn	10.00	0.42 ± 0.03	3.77 ± 0.27	—
	Xyl43	0.25	0.02 ± 0.01	0.21 ± 0.09	—
	Xyl43	1.00	0.05 ± 0.01	0.47 ± 0.09	—
	Xyn + Xyl43	10.00 + 0.25	0.81 ± 0.10	7.19 ± 0.89	1.81
	Xyn + Xyl43	10.00 + 1.00	1.33 ± 0.06	11.81 ± 0.54	2.76
	RE-10	10.00	0.33 ± 0.12	2.91 ± 1.08	—
	RE-10 + Xyl43	10.00 + 0.25	1.43 ± 0.03	12.71 ± 0.27	3.96
	RE-10 + Xyl43	10.00 + 1.00	1.86 ± 0.13	16.53 ± 1.16	4.89

^a^Hydrolysis condtions: 50 °C, pH 7.0 for 24 h.

## Conclusions

A novel GH 43 β-xylosidase from *P*. *oxalicum* was high-level expressed using the expression system of *P*. *pastoris* X-33 for secreting more protein and producing high activity β-xylosidase. The recombinant Xyl43 is high xylose tolerant and high pH tolerant, and exhibits high catalytic activity in the range of pH 6.0 to 8.0. The Xyl43 could effectively enhance the enzymatic hydrolysis of different sources xylan and alkaline pretreated corn stover by synergistic effect with xylanase for producing high-yield xylose, and showed a good potential in bioconversion of biomass containing more hemicellulose for production of bioethanol and chemicals.

## Materials and Methods

### Strains and plasmids

*P*. *oxalicum* 114-2 and *P*. *oxalicum* RE-10 were stored in our laboratroy. *E*. *coli* DH5α and pEASY-Blunt Zero vector from TransGen (Beijing, China) were used for propagation of plasmids and gene clone. The plasmid pPICZαA and host *P*. *pastoris* X-33 from Invitrogen (Carlsbad, CA, USA) were employed for the β-xylosidase gene expression. All strains and plasmids used in this study shown in supplementary Table S3.

### Chemicals and enzyme

The chemicals as substrates for enzyme activities assay, including *p*-Nitrophenyl-β-D-glucopyranoside (*p*NPG), *p*-Nitrophenyl-β-D-xyloside (*p*NPX), *p*-Nitrophenyl-L-arabinofuranoside (*p*NPAf), *p*-Nitrophenyl-D-cellobioside (*p*NPC) and beechwood xylan, were purchased from Sigma (St. Louis, USA). Corncob xylan and sugarcane bagasse xylan were obtained from Yuanye (Shanghai, China).

Crude xylanase RE-10 was produced using engineered strain *P*. *oxalicum* RE-10 according to the method reported in literature^40^. It has xylanase activity of 10 IU/ml and β-xylosidase activity of 0.09 IU/ml, but ignored filter paper activity (FPA) under neutral condition (pH 7.0).

### Extraction of mRNA and PCR cloning of β-xylosidase gene *xyl43*

*P*. *oxalicum* 114-2 was cultured for mRNA extraction as described previously^40^. Total RNA was isolated from the mycelia of *P*. *oxalicum*114-2 after 24 h growth in the medium with 2% wheat bran inducer. The cDNA synthesis using Prime Script RT Reagent Kit (TaKaRa, Japan) was performed according to the manufacturer’s instructions. The gene fragment of *xyl43* was amplified by PCR from the cDNA as template using primers xyl-f and xyl-r (see supplementary Table S4). The PCR products were purified using 1% (w/v) agarose gel electrophoresis, then ligated into pEASY-Blunt Zero vector. The pEASY-*xyl43* recombinant plasmid was extracted for nucleotide sequencing. The gene fragment of *xyl43* containing the homologous sequences region with pPICZαA vector was amplified by PCR from positive vector pEASY-*xyl43* as template using primers xyl-F and xyl-R (see supplementary Table S4), then ligated into the plasmid pPICZαA digested by *EcoR*I and *Not*I, using ClonExpress^®^ II One Step Cloning Kit (Vazyme, Nanjing, China) for recombination according to the manufacturer’s protocols.

### Expression, production and purification of recombinant β-xylosidase and endo-xylanase

Mix 100 μl of *P*. *pastoris* X-33 competent cells with 20 μg of linearized recombinant plasmid pPICZαA-*xyl43* by *Sac*Ι and transferred them to 0.2 cm electroporation cuvette for electroporation using Gene Pulser Xcell^TM^ Electroporation System (Bio-Rad, Hercules, CA). YPDS solid culture medium (1% yeast extract, 2% peptone, 2% glucose, 1 M sorbitol, 2% agar) containing 100 μg/ml Zeocin were prepared for screening of positive transformants. *P*. *pastoris* X-33 transformants were cultured on BMGY medium (1% yeast extract, 2% peptone, 100 mM potassium phosphate pH 6.0, 1.34% Yeast Nitrogen Base, 0.4 μg/ml Biotin and 1% glycerol) at 30 °C in a shaking incubator at 200 rpm for 12 h to16 h. The cells were collected by centrifugation at 5000× *g* for 10 min at 4 °C and subsequently were subcultured on BMMY medium (1% yeast extract, 2% peptone, 100 mM potassium phosphate pH 6.0, 1.34% Yeast Nitrogen Base, 0.4 μg/ml Biotin and 1% methanol) at 30 °C in a shake flask of 200 rpm for expressing recombinant enzyme.

The recombinant enzyme containing a C-terminal His6tag was purified using a HisTrap^TM^FF column (Histrap, GE Healthcare, USA). The recombinant β-xylosidase (Xyl43) molecular weight was determined by 12.5% SDS-PAGE and stained with Coomassie brilliant R-250. The target band of the Xyl43 was excised and submitted for MALDI-TOF analysis (APT, Shanghai, China). Deglycosylation of Xyl43 was performed using Endoglycosidase H (Endo H, New England BioLabs, MA) according to the protocol provided by manufacturer. The deglycosylated enzyme was also analyzed by SDS-PAGE. Protein concentration was determined according to the Bradford method^41^.

The endo-xylanase (Xyn) from *P*. *oxalicum* 114-2 was also expressed in *P*. *pastoris* GS115 and purified using a HisTrap^TM^ FF column. The purified Xyn has a specific activity of 113.42 IU/ml.

### Enzymatic characterization of recombinant Xyl43

#### Optimal pH and stability on pH value

Optimal pH of the purified recombinant Xyl43 was determined by measuring the enzyme activity in different pH values (pH 3.0 to 10.0) according to enzyme activity assay method. The pH of reaction system was adjusted by following buffers: 50 mM Na_2_HPO_4_-citric acid (pH 2.0-8.0), 50 mM Tris-HCl (pH 8.0–9.0) and 50 mM glycine-NaOH (pH 9.0–11.0), respectively. The stability of the purified Xyl43 on pH values was studied by incubating in different pH buffers as mentioned above at 30 °C for 60 min, followed by residual activity determination at 50 °C according to activity assay method.

#### Optimal temperature and thermo-stability

Optimal temperature of the Xyl43 was conducted by measuring β-xylosidase activity in different temperature (30 °C to 80 °C) at the optimal pH according to enzyme activity assay method. For thermo-stability analysis, the purified β-xylosidase in 50 mM phosphate buffer (pH 7.0) was incubated at temperature of 30 °C to 80 °C for 30 min, respectively. After 30 min, the samples were rapidly cooled on ice-water bath and the residual activity was measured.

#### Effect of metal ions, chemical reagents and monosaccharide sugars on enzyme activity

To evaluate the effects of metal ions, chemical reagents and monosaccharide sugars on the enzyme activity, 5 mM of various metal ions or chemical reagents (K^+^, Cu^2+^, Mn^2+^, Ca^2+^, Pb^2+^, Co^2+^, Zn^2+^, Mg^2+^, Fe^3+^, Ba^2+^, SDS, EDTA) and 20 mM of different monosaccharide sugars (glucose, mannose, galactose, arabinose, and xylose) were added to reaction system, respectively. The control was done according to same process without above any additive in reaction mixture.

#### Effect of xylose concentrations on enzyme activity

To investigate the effect of end product xylose on catalytic activity, reaction were carried out in the presence of various xylose concentrations of 1 mM to 67 mM, in which, the substrate *p*NPX concentrations were 0.5 mg/ml and 1 mg/ml, respectively. Same reaction system, but no adding xylose, was used as the control.

#### Determination of substrate specificity

To determine the substrate specificity of β-xylosidase Xyl43, different *p*-nitrophenyl derivatives such as *p*NPG, *p*NPAf and *p*NPC, and beechwood xylan, glucan, mannan, polygalactose and arabinan, were used as substrates for enzyme activity measure.

#### Determination of kinetic constants

Kinetic constants (Vmax and Km) of Xyl43 were performed with different concentrations of *p*NPX (0.5 mg/ml to 5.0 mg/ml) at 50 °C and pH 7.0 for 5 min, in presence of a fixed inhibitor concentration of 10 mM xylose, or not. The values of Vmax and Km of the xylosidase for *p*NPX were calculated using Lineweaver-Burk plots.

### Enzymatic hydrolysis

The enzymatic hydrolysis of different xylans such as beechwood xylan, corncob xylan and sugarcane bagasse xylan were performed by adding appropriate pure Xyn and/or Xyl43 in a 1.5 ml of reaction system containing 50 mM sodium phosphate buffer (pH 7.0), 0.5 ml of 1% (w/v) xylan at 50 °C for 24 h. The dosage of Xyl43 and Xyn was 0.05 IU/mg and 0.2 IU/mg based on dry weight of xylan.

Alkaline pretreatment of corn stover was carried out according to the method as described in the literatures^42, 43^. The APCS were firstly made by adding corn stover (6.03% moisture) to 1% NaOH solution, the ratio of corn stover to solution was 6:1 (w/v), then treated at 170 °C for 1 h. The pretreated corn stover was repeatedly washed by tap water to remove residual NaOH, and stored at 4 °C for experimental usage.

The enzymatic hydrolysis of APCS was conducted by Xyl43 with and without Xyn or RE-10 in 50 ml of sodium phosphate buffer (pH 7.0) at 50 °C for 24 h. The APCS consistency was 5% (w/v), the dosage of Xyl43 was 0.25 IU/g and 1.0 IU/g respectively based on β-xylosidase activity, and the dosages of Xyn and RE-10 were 10 IU/g and 10 IU/g based on xylanase activity and dry APCS weight.

After enzymatic hydrolysis, the reactions were terminated by boiling for 10 min. The supernatants were collected by centrifugation at 4 °C, 12000 × *g* for 10 min, and then passed through a 0.22 μm filter to remove impurities. The xylose contents in hydrolysis liquors were measured by high performance liquid chromatography (HPLC) with the refractive index detector in a Bio-Rad AminexHPX-87P column using water for elution at flow rate 0.5 ml/min at 78 °C.

### Analysis methods

#### Assays of enzyme activities

Assay of β-xylosidase activity was performed as described previously^44^ using *p*NPX as substrate.The reaction mixture contained 50 μl of 1.0 mg/ml *p*NPX (dissolved in 50 mM sodium phosphate buffer, pH 7.0) and 100 μl of appropriately diluted enzyme. After incubated at 50 °C for 30 min, the reaction system was stopped by adding150 μl of 1 M Na_2_CO_3_. One unit of enzyme activity was defined as the amount of enzyme that produced 1 μmol of *p*-nitrophenol per minute under assay conditions.

Xylanase activity was determined using beechwood xylan as the substrate according to the dinitrosalicylic acid (DNS) method^45^. The enzyme sample (0.5 ml) was incubated with 1.5 ml of the 1% beechwood xylan in 50 mM citrate-phosphate buffer (pH 7.0) at 50 °C for 30 min.

Filter paper activity (FPA) was estimated as reported in literature^46^. The reaction mixture included 0.5 ml of enzyme sample and 1.5 ml of 50 mM citrate buffer (pH 7.0), and assay was performed at 50 °C for 60 min using Whatman No. 1 filter paper (50 ± 0.5 mg, 1 × 6 cm) as the substrate.

The reducing sugars were measured by adding 3 ml of DNS, boiling for 10 min and detecting the absorbance at 540 nm. One enzyme activity unit was defined as the amount of enzyme required to release 1 μmol xylose or glucose per minute under the measure conditions.

#### Chemical components analysis

The chemical components of APCS were measured by a two-stage sulfuric acid hydrolysis treatment according to the NREL laboratory analytical method^47^.

#### Statistical analysis

All experiments were conducted in triplicate. The mean values, standard deviations were calculated in the quantitative analysis using the Microsoft Office 2010 Excel software in this work. The value of enzyme activity was indicated the mean, and other values were showed the mean ± standard deviations (SD; n = 3). Statistical tests were conducted with one-tail *t*-Student tests by software Microsoft Excel 2007.

Conversion of xylan to xylose was calculated according to the formula:

$$\begin{array}{c}{\rm{Conversion}}\,( \% )=\frac{{\rm{Total}}\,{\rm{xylose}}\,{\rm{released}}\,{\rm{amount}}\,(\mathrm{mg})\,{\rm{by}}\,{\rm{enzymatic}}\,{\rm{hydrolysis}}\,\times \,{\rm{0.88}}}{{\rm{The}}\,{\rm{amount}}\,(\mathrm{mg})\,{\rm{of}}\,{\rm{xylan}}\,{\rm{in}}\,{\rm{substrate}}}\times \mathrm{100} \% \end{array}$$

Synergy factor is designed as the ratio of amount of xylose released by Xyl43 and Xyn (or RE-10) to the sum of amount of xylose released by only Xyl43 and only Xyn (or RE-10) respectively.

## Electronic supplementary material

Supplementary figures and Tables

## References

[1] Girio FM (2010). Hemicelluloses for fuel ethanol: A review. Bioresour. Technol..

[2] Lei Z (2016). Combination of Xylanase and Debranching Enzymes Specific to Wheat Arabinoxylan Improve the Growth Performance and Gut Health of Broilers. J. Agric. Food Chem..

[3] Tanaka T, Hirata Y, Nakano M, Kawabata H, Kondo A (2014). Creation of cellobiose and xylooligosaccharides-coutilizing *Escherichia coli* displaying both beta-glucosidase and beta-xylosidase on its cell surface. ACS Synth Biol..

[4] Qing Q, Wyman CE (2011). Hydrolysis of different chain length xylooliogmers by cellulase and hemicellulase. Bioresour. Technol..

[5] Dien BS (2008). Enzyme characterization for hydrolysis of AFEX and liquid hot-water pretreated distillers’ grains and their conversion to ethanol. Bioresour. Technol..

[6] Yang X (2014). Two xylose-tolerant GH43 bifunctional beta-xylosidase/alpha-arabinosidases and one GH11 xylanase from *Humicola insolens* and their synergy in the degradation of xylan. Food Chem..

[7] Mustafa G, Kousar S, Rajoka MI, Jamil A (2016). Molecular cloning and comparative sequence analysis of fungal beta-Xylosidases. AMB. Express..

[8] Brüx C (2006). The structure of an inverting GH43 β-Xylosidase from *Geobacillus stearothermophilus* with its substrate reveals the role of the three catalytic residues. J. Mol. Biol..

[9] Shallom D (2005). Biochemical characterization and identification of the catalytic residues of a family 43 beta-D-xylosidase from *Geobacillus stearothermophilus* T-6. Biochemistry..

[10] Honda Y (2008). Alternative strategy for converting an inverting glycoside hydrolase into a glycosynthase. Glycobiology..

[11] Knob A, Terrasan C, Carmona E (2010). β-Xylosidases from filamentous fungi: an overview. World J. Microbiol. Biotechnol..

[12] Jordan DB, Wagschal K, Grigorescu AA, Braker JD (2013). Highly active beta-xylosidases of glycoside hydrolase family 43 operating on natural and artificial substrates. Appl. Microbiol. Biotechnol..

[13] Chen Z (2012). Secretory expression of a beta-xylosidase gene from *Thermomyces lanuginosus* in *Escherichia coli* and characterization of its recombinant enzyme. Lett. Appl. Microbiol..

[14] Yan Q (2008). A xylose-tolerant β-xylosidase from *Paecilomyces thermophila*: characterization and its co-action with the endogenous xylanase. Bioresour. Technol..

[15] Sommer B, Friehs K, Flaschel E (2010). Efficient production of extracellular proteins with *Escherichia coli* by means of optimized coexpression of bacteriocin release proteins. J. Biotechnol..

[16] Ni Y, Chen R (2009). Extracellular recombinant protein production from *Escherichia coli*. Biotechnol. Lett..

[17] Juturu V, Wu JC (2013). Heterologous expression of beta-xylosidase gene from *Paecilomyces thermophila* in *Pichia pastoris*. World J. Microbiol. Biotechnol..

[18] Yinbo Q, Xin Z, Peiji G, Zunong W (1991). Cellulase production from spent sulfite liquor and paper-mill waste fiber. Appl. Biochem. Biotechnol..

[19] Ravanal MC, Alegria-Arcos M, Gonzalez-Nilo FD, Eyzaguirre J (2013). *Penicillium purpurogenum* produces two GH family 43 enzymes with beta-xylosidase activity, one monofunctional and the other bifunctional: Biochemical and structural analyses explain the difference. Arch. Biochem. Biophys..

[20] Teng C, Jia H, Yan Q, Zhou P, Jiang Z (2011). High-level expression of extracellular secretion of a beta-xylosidase gene from *Paecilomyces thermophila* in *Escherichia coli*. Bioresour. Technol..

[21] Wongwisansri S (2013). High-level production of thermotolerant beta-xylosidase of *Aspergillus sp*. BCC125 in *Pichia pastoris*: characterization and its application in ethanol production. Bioresour. Technol..

[22] Huy ND, Thayumanavan P, Kwon TH, Park SM (2013). Characterization of a recombinant bifunctional xylosidase/arabinofuranosidase from *Phanerochaete chrysosporium*. J. Biosci. Bioeng..

[23] Lee CC, Braker JD, Grigorescu AA, Wagschal K, Jordan DB (2013). Divalent metal activation of a GH43 beta-xylosidase. Enzyme Microb. Technol..

[24] Kirikyali N, Connerton IF (2014). Heterologous expression and kinetic characterisation of *Neurospora crassa* beta-xylosidase in *Pichia pastoris*. Enzyme Microb. Technol..

[25] Kirikyali N, Wood J, Connerton IF (2014). Characterisation of a recombinant β-xylosidase (xylA) from *Aspergillus oryzae* expressed in *Pichia pastoris*. AMB. Express..

[26] Xia W (2015). High level expression of a novel family 3 neutral beta-xylosidase from *Humicola insolens* Y1 with high tolerance to D-xylose. PLoS One..

[27] Ito T (2003). Xylosidases associated with the cell surface of *Penicillium herquei* IFO 4674. J. Biosci. Bioeng..

[28] Huy ND, Nguyen CL, Seo JW, Kim DH, Park SM (2015). Putative endoglucanase PcGH5 from *Phanerochaete chrysosporium* is a beta-xylosidase that cleaves xylans in synergistic action with endo-xylanase. J. Biosci. Bioeng..

[29] Kumar V, Satyanarayana T (2013). Biochemical and thermodynamic characteristics of thermo-alkali-stable xylanase from a novel polyextremophilic *Bacillus halodurans* TSEV1. Extremophiles..

[30] Suzuki S (2010). Characterization of *Aspergillus oryzae* glycoside hydrolase family 43 beta-xylosidase expressed in *Escherichia coli*. J. Biosci. Bioeng..

[31] Kim YA, Yoon K-H (2010). Characterization of a *Paenibacillus woosongensis* β-xylosidase/α-arabinofuranosidase produced by recombinant *Escherichia coli*. J. Microbiol. Biotechnol..

[32] Rasmussen LE, Sorensen HR, Vind J, Vikso-Nielsen A (2006). Mode of action and properties of the beta-xylosidases from *Talaromyces emersonii* and *Trichoderma reesei*. Biotechnol. Bioeng..

[33] Knob A, Carmona EC (2009). Cell-associated acid beta-xylosidase production by *Penicillium sclerotiorum*. N. Biotechnol..

[34] La Grange DC, Pretorius IS, Claeyssens M, van Zyl WH (2001). Degradation of xylan to D-xylose by recombinant *Saccharomyces cerevisiae* coexpressing the *Aspergillus niger* beta-xylosidase (xlnD) and the *Trichoderma reesei* xylanase II (xyn2) genes. Appl. Environ. Microbiol..

[35] Kumar S, Ramón D (1996). Purification and regulation of the synthesis of a β‐xylosidase from *Aspergillus nidulans*. FEMS. Microbiol. Lett..

[36] Jain I, Kumar V, Satyanarayana T (2014). Applicability of recombinant beta-xylosidase from the extremely thermophilic bacterium *Geobacillus thermodenitrificans* in synthesizing alkylxylosides. Bioresour. Technol..

[37] Hayashi S, Ohno T, Ito M, Yokoi H (2001). Purification and properties of the cell-associated β-xylosidase from. Aureobasidium. J. Ind. Microbiol. Biotechnol..

[38] Kirikyali N, Connerton IF (2015). Xylan degrading enzymes from fungal sources. J. Proteomics & Enzymology..

[39] Lagaert S, Pollet A, Courtin CM, Volckaert G (2014). beta-xylosidases and alpha-L-arabinofuranosidases: accessory enzymes for arabinoxylan degradation. Biotechnol. Adv..

[40] Yao G (2015). Redesigning the regulatory pathway to enhance cellulase production in *Penicillium oxalicum*. Biotechnol. Biofuels..

[41] Bradford MM (1976). A rapid and sensitive method for the quantitation of microgram quantities of protein utilizing the principle of protein-dye binding. Anal. Biochem..

[42] Ye C, Stevens MA, Zhu Y (2015). Understanding of alkaline pretreatment parameters for corn stover enzymatic saccharification. Biotechnol. Biofuels..

[43] Hu J, Zhang Z, Lin Y (2015). High-titer lactic acid production from NaOH-pretreated corn stover by *Bacillus coagulans*, LA204 using fed-batch simultaneous saccharification and fermentation under non-sterile condition. Bioresour. Technol..

[44] Diogo JA (2015). Development of a chimeric hemicellulase to enhance the xylose production and thermotolerance. Enzyme Microb. Technol..

[45] Bailey MJ, Biely P, Poutanen K (1992). Interlaboratory testing of methods for assay of xylanase activity. J. Biotechnol..

[46] Saini R (2015). Enhanced cellulase production by *Penicillium oxalicum* for bio-ethanol application. Bioresour. Technol..

[47] Sluiter, A. *et al*. Determination of structural carbohydrates and lignin in biomass. http://purl.access.gpo.gov/GPO/LPS94089 (Date of access: 4/25/2008) (2008).

[48] Gramany, V. *et al*. Cloning, expression, and molecular dynamics simulations of a xylosidase obtained from *Thermomyces lanuginosus*. *J*. *Biomol*. *Struct*. *Dyn*. 1–12 (2015). 10.1080/07391102.2015.1089186 26336893

[49] Ravanal MC, Callegari E, Eyzaguirre J (2010). Novel bifunctional alpha-L-arabinofuranosidase/xylobiohydrolase (ABF3) from *Penicillium purpurogenum*. Appl. Environ. Microbiol..

[50] Poutanen K, Puls J (1988). Characteristics of *Trichoderma reesei* β-xylosidase and its use in the hydrolysis of solubilized xylans. Appl. Environ. Microbiol..

