# Supplementary material for: Production and Characteristics of a Novel Xylose- and Alkali-tolerant GH 43 β-xylosidase from Penicillium oxalicum for Promoting Hemicellulose Degradation
Source: Sci Rep. 2017 Sep 14;7:11600. doi: 10.1038/s41598-017-11573-7 (PMC5599605; doi:10.1038/s41598-017-11573-7)
Supplement: Supplementary file 1 — Supplementary figures and Tables [file 41598_2017_11573_MOESM1_ESM.pdf]

Production and Characteristics of a Novel Xylose- and Alkali-tolerant GH 43 β-xylosidase from *Penicillium oxalicum* for Promoting Hemicellulose Degradation

Yanxin Ye, Xuezhi Li, Jian Zhao*

State Key Laboratory of Microbial Technology, Shandong University, Jinan, 250100, P.R. China

*Corresponding author: Jian Zhao. Tel.: +86-531-88364690, Fax: +86-531-88565234, Email: zhaojian@sdu.edu.cn

Supplementary Fig. S1. Sequence alignment results of Xyl43 from *P. oxalicum* with other GH43 β-xylosidases by ClustalX/W software and secondary structure of the Xyl43 validated by i-TASSER alignment. Identical and similar residues are shaded in black and grey, respectively.

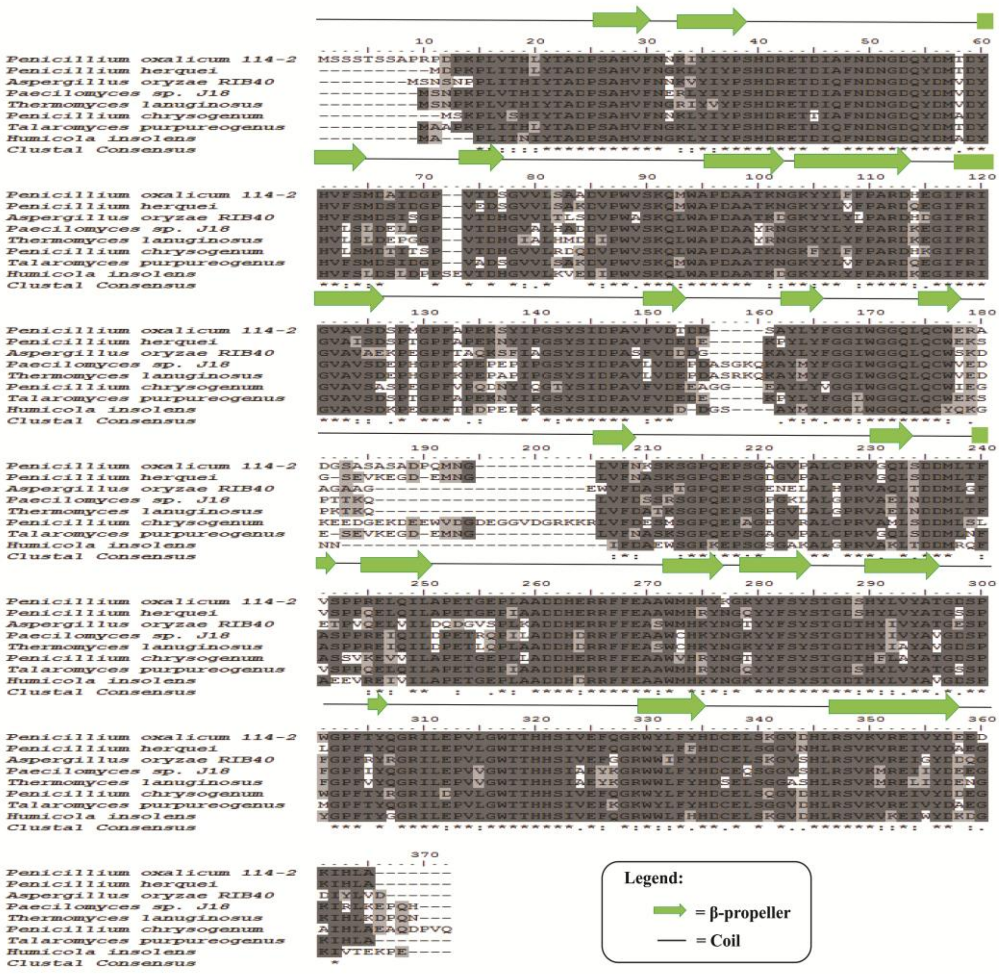

Supplementary Fig. S2. The copy numbers of GH43 β-xylosidase gene by southern blotting analysis. Only one band was appeared in the genomic DNA of single-copy strain (green arrows). Other bands in multi-copy strain (red arrows) indicated that multiple β-xylosidase gene *xyl3A* copies were inserted into the genomic DNA.

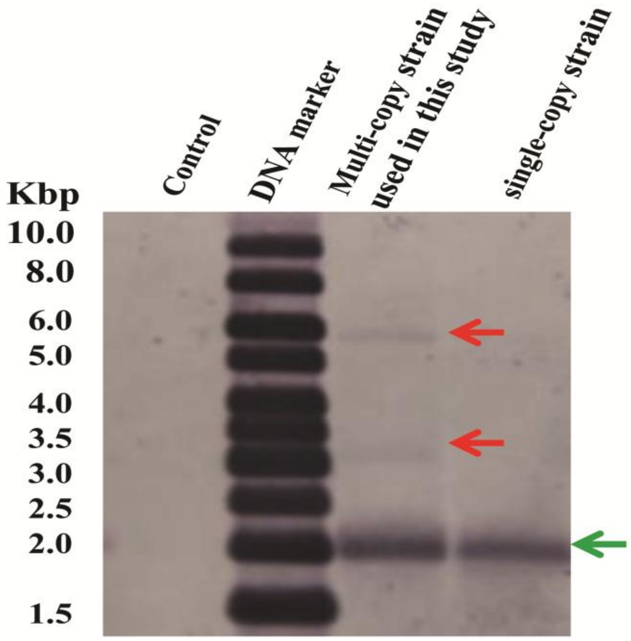

Supplementary Fig. S3. Production profile of Xyl43 expressed in *P. pastoris*.

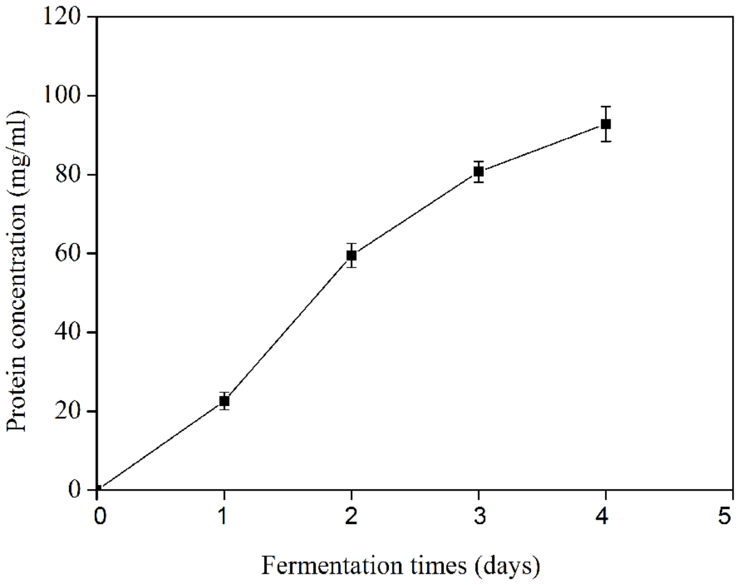

Supplementary Fig. S4. SDS-PAGE analysis of Xyl43. Lane M, protein marker; Lane 5, Xyl43 after Endo H treatment; Lane 6, purified Xyl43.

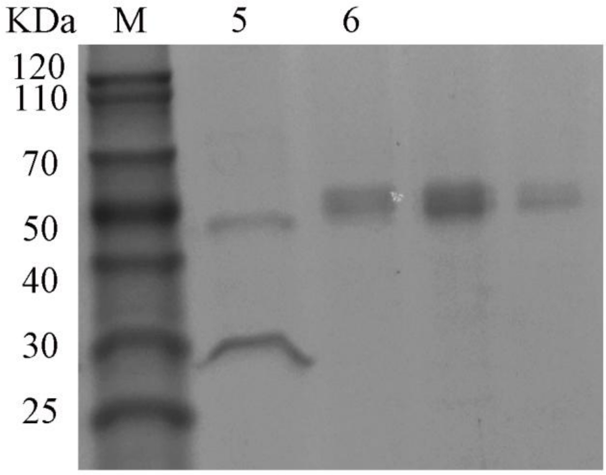

Supplementary Table S1

Predicted potential N-glycosylation/O-glycosylation sites in the Xyl43 sequence.

Position	Amino acid	N-glycosylation	O-glycosylation
191	Asn	Positive	-
2	Ser	-	Positive
4	Thr	-	Positive
5	Ser	-	Positive
6	Ser	-	Positive
7	Ser	-	Positive
180	Ser	-	Positive
201	Ser	-	Positive

Supplementary Table S2

Alignment of secondary structure of Xyl43 with other known structure of GH43 enzymes by i-TASSER analysis.

PDB code	Sequence identity (100%)	E-value
5gllA	57.0	4.59e-113
3qeeA	30.0	9.65e-29
4mlgA	54.0	1.09e-103

Supplementary Table S3

Strains and plasmids used in this study.

Strains or plasmids	Function	Source
**Strains**		
P. oxalicum 114-2	mRNA extraction	Our laboratory
E. coli DH5α	Host for *xyl43* cloning	TransGen (Bei jing, China)
P. Pastoris X-33	Host for *xyl43* expression	Invitrogen (Carlsbad, CA, USA)
P. oxalicum RE-10	Xylanase complex production	Our laboratory
Recombinant *P. pastoris* GS115	Xylanase production	Our laboratory
**plasmids**		
pEASY-Blunt Zero	Cloning vector	TransGen (Bei jing, China)
pPICZαA	Expression vector	Invitrogen (Carlsbad, CA, USA)

Supplementary Table S4

The specific primers used for this work.

Primers	Primer sequence (5′ →3′)[a]	Size (bp)
xyl-f	TCTTCTTCTACTTCCTCGGCGC	22
xyl-r	TGCCAGATGAATCTTATCCTCCTC	24
xyl-F	GAGAGGCTGAAGCT*GAATTC*TCTTCTTCTACTTCCTCGGCGC	42
xyl-R	TCTAGAAAGCTG*GCGGCCGC*TGCCAGATGAATCTTATCCTCCTC	44

[a] The homologous sequences region with pPICZαA vector incorporated into primers are shown underlined and restriction sites in italics.
